# Clinical Efficacy and Safety of Cox-Maze IV Procedure for Atrial Fibrillation in Patients With Hypertrophic Obstructive Cardiomyopathy

**DOI:** 10.3389/fcvm.2021.720950

**Published:** 2021-08-02

**Authors:** Yanhai Meng, Yanbo Zhang, Ping Liu, Changsheng Zhu, Tao Lu, Enci Hu, Qiulan Yang, Changrong Nie, Shuiyun Wang

**Affiliations:** ^1^Surgery Intensive Care Unit, Department of Cardiovascular Surgery, Fuwai Hospital, National Center for Cardiovascular Diseases, Chinese Academy of Medical Sciences and Peking Union Medical College, Beijing, China; ^2^Adult Cardiac Surgery, Department of Cardiovascular Surgery, Fuwai Hospital, National Center for Cardiovascular Diseases, Chinese Academy of Medical Sciences and Peking Union Medical College, Beijing, China

**Keywords:** hypertrophic cardiomyopathy, obstructive, atrial fibrillation, surgical ablation, efficacy, safety

## Abstract

**Objective:** Atrial fibrillation is the most prevalent persistent arrhythmia in patients with hypertrophic obstructive cardiomyopathy. Comparative analyses of the safety and effectiveness of septal myectomy with and without surgical ablation are limited. This study aimed to compare the outcomes of septal myectomy with and without the Cox-maze IV procedure in patients with hypertrophic obstructive cardiomyopathy and atrial fibrillation.

**Methods:** Ninety-four patients with hypertrophic obstructive cardiomyopathy and atrial fibrillation who underwent septal myectomy were analyzed, we divided it into concomitant Cox maze surgery (Cox-maze group) and no concomitant Cox maze operation (no Cox-maze group). Freedom from atrial fibrillation recurrence and all-cause mortality after surgery were assessed.

**Results:** Freedom from all-cause mortality after septal myectomy at 1, 3, and 5 years was 98.5 ± 1.5% each in the Cox-maze group and 90.8 ± 6.3%, 85.1 ± 8.1%, and 85.1 ± 8.1%, respectively, in the no Cox-maze group. Patients in the no Cox-maze group had lower survival, freedom from atrial fibrillation recurrence off antiarrhythmic drugs, and arrhythmia control rate (including patients with successful antiarrhythmic drug conversion) than those in the Cox-maze group (*P* = 0.046, *P* = 0.040, and *P* = 0.012, respectively). Patients who underwent the Cox-maze IV procedure had lower atrial fibrillation recurrence rate than those who did not (hazard ratio, 0.141; 95% confidence interval, 0.042–0.479; *P* = 0.002). Post-operative increases in left atrial diameters (hazard ratio, 1.099; 95% confidence interval, 1.024–1.179; *P* = 0.009) were associated with atrial fibrillation recurrence.

**Conclusions:** The Cox-maze IV procedure combined with septal myectomy improved mid-term survival and reduced mid-term atrial fibrillation recurrence in patients with hypertrophic obstructive cardiomyopathy and atrial fibrillation. The concomitant Cox-maze IV procedure was associated with a lower atrial fibrillation recurrence in patients with surgical hypertrophic obstructive cardiomyopathy and atrial fibrillation.

## Introduction

Hypertrophic obstructive cardiomyopathy (HOCM) caused by left ventricular outflow tract (LVOT) obstruction is the most frequent type of hypertrophic cardiomyopathy (HCM) (1), and septal myectomy is the gold standard for the treatment of HOCM (2). Atrial fibrillation (AF) is the most common persistent arrhythmia in patients with HCM (3), with an incidence between 18 and 28% (4). Patients with HOCM have poor tolerance to AF, which leads to frequent admissions and poor quality of life (5). Mitral regurgitation due to systolic anterior motion (SAM), LVOT obstruction, and particularly left ventricular (LV) diastolic dysfunction, which result in increased left atrial pressure and chronic atrial remodeling, may be responsible for the increased vulnerability to initiate and maintain AF in HOCM (3).

For patients with HOCM complicated with AF, dealing with AF is extremely difficult. Pharmacologic therapy has a limited effect on restoring sinus rhythm (SR) (6). Although catheter ablation is effective, the recurrence rate of AF remains high, ranging from 33 to 55% after an isolated ablation procedure (7). In recent years, due to the recognition of the serious harm of AF in patients with HOCM (8) and the development of surgical ablation (9), a few studies have reported the results of surgical ablation in patients with HOCM and AF (10–12). However, comparisons of the safety and effectiveness of septal myectomy with and without ablation are limited. Therefore, this study aimed to retrospectively analyse the differences in AF recurrence and mid-term follow-up outcomes between septal myectomy with and without surgical ablation in patients with HOCM and AF.

## Patients and Methods

Due to concerns about the success rate and safety of the Cox-maze procedure, as well as the cost of the operation, some patients did not undergo the Cox-maze procedure at the same time as septal myectomy, and we retrospectively analyzed the post-operative and follow-up results. Details on the diagnostic criteria of HOCM, AF, and AF recurrence are presented in the Supplementary Material 1.

A total of 94 consecutive patients with HOCM who underwent septal myectomy with and without the Cox-maze procedure (we divided them into the Cox-maze group and no Cox-maze group) at our institution between January 2015 and July 2020 were assessed. For patients in Cox-maze group, most patients underwent Cox-maze IV operation (*n* = 54), and a small number of patients underwent left atrial ablation operation (*n* = 14). This study was approved by the ethics committee of Fuwai Hospital (number of approval: 2020-1277). This study was conducted in compliance with the principles of the Declaration of Helsinki.

### Surgical Technique

We performed an extended septal myectomy, as described previously (13). Details are presented in the Supplementary Material 2. Ablation lines of Cox-maze IV procedure were created using an Isolator Synergy bipolar device (AtriCure, Inc., Cincinnati, OH) applied six times at each line. The ablation route was based on the classic Cox-maze IV procedure (14), and details are presented in the Supplementary Material 3.

### Post-operative Monitoring and Follow-Up

The patients received intravenous infusion of amiodarone at a total dose of 1,200 mg within 24 h after the surgery (amiodarone was stopped when autonomic heart rate was lower than 50 beats/min); subsequently, they were administered amiodarone 200 mg orally, three times a day for 7 days, twice a day for 7 days, and then maintained once a day for 3 months. Thyroid function was monitored regularly during medication administration. Heparin (10–20 mg, q6h) was administered to prevent thrombosis when pericardial/mediastinal drainage decreased 4–6 h after the operation. Warfarin was administered through the gastric tube at the same time. Heparin was stopped when the international normalized ratio was 2.0–2.5. Warfarin anticoagulant therapy was administered for 6 months and was stopped if there was no AF recurrence.

All patients were followed up at the time of discharge, 6 months, 1 year, and yearly thereafter, with physical examination, 12-lead ECG, 24-h Holter monitoring, and echocardiography. We defined the patients whose arrhythmias were controlled as those who maintained sinus rate and those who were successfully converted with drugs after AF recurrence after surgery. The criteria for successful conversion and maintenance of SR were defined as definite SR on 12-lead precordial ECG, and no AF was found in 24-h Holter results. The primary end point of this study was comparison of freedom from atrial arrhythmia recurrence and all-cause mortality between the two groups, atrial arrhythmia recurrence defined as any documented AF, atrial flutter, or atrial tachycardia episode of >30 s after a 3-month blanking period.

### Statistical Analyses

Normal distribution of continuous variables was assessed by Kolmogorov–Smirnov test. Continuous data are expressed as means ± standard deviations or medians [interquartile ranges (IQRs)] depending on whether the data is normally distributed. These data were compared using Student's *t*-test or the Mann–Whitney *U-*test depending on whether the data is normally distributed. Categorical data are presented as numbers (percentages) and were compared using the chi-squared test or Fisher's exact test as appropriate. The Kaplan–Meier method was used with log-rank tests to evaluate event-free survival. A Cox proportional hazards regression model was used to adjust for confounding factors. Age, sex, and variables with *P* < 0.1 in the univariable analysis were included in the multivariate regression model. A two-sided *P* < 0.05 was considered statistically significant. Statistical analyses were performed using IBM^®^ SPSS^®^ version 22.0 (IBM Corp., Armonk, NY, USA).

## Results

### Patient Characteristics

A total of 94 consecutive patients were enrolled in the study. There were 54 men (57.45%), 42 patients with persistent AF (44.68%), and 52 patients with paroxysmal AF (55.32%). The median (IQR) age was 53 (42.75, 62) years, and most patients (72/94) had New York Heart Association (NYHA) functional class ≥III. The baseline clinical characteristics of the study population are shown in Table 1. Compared with patients in the no Cox-maze group, those in the Cox-maze group had higher EuroSCORE (2.84 ± 1.00 vs. 1.77 ± 1.42 mm, *P* = 0.001) and were more likely to have dyspnoea (92.65 vs. 76.92%, *P* = 0.034).

**Table 1:** Baseline characteristics and pre-operative clinical variables of patients in the Cox-maze and no Cox-maze group (Cox-maze *N* = 68).

**Characteristics**	**Cox-maze (*N* = 54) (%)**	**No Cox-maze (*N* = 26) (%)**	***P*-value**
Age (year), median (IQR)	53 (42, 61)	54 (42, 64)	0.729
Gender (males), *n* (%)	43 (63.23)	11 (42.31)	0.066
Body mass index (kg/m^2^), mean ± SD	26.22 ± 3.36	26.39 ± 5.15	0.856
NYHA functional class III/IV, *n* (%)	52 (76.47)	20 (76.92)	1.00
EuroSCORE II, mean ± SD	2.84 ± 1.00	1.77 ± 1.42	0.001
Paroxysmal, *n* (%)	36 (52.94)	16 (61.54)	0.453
**Symptoms**			
Dyspnoea, *n* (%)	63 (92.65)	20 (76.92)	0.034
Chest pain, *n* (%)	18 (26.47)	11 (42.31)	0.137
Syncope, *n* (%)	17 (25)	2 (7.69)	0.062
Palpitation, *n* (%)	20 (29.41)	8 (30.77)	0.898
**Comorbidities**			
Hypertension, *n* (%)	25 (36.76)	7 (26.92)	0.368
Hyperlipidemia, *n* (%)	12 (17.65)	9 (34.62)	0.077
Diabetes mellitus, *n* (%)	6 (8.82)	1 (3.85)	0.411
Coronary atherosclerotic heart disease, *n* (%)	6 (8.81)	1 (3.85)	0.411
Myocardial bridge, *n* (%)	3 (4.41)	4 (15.38)	0.070
History of cerebral infarction, *n* (%)	3 (4.41)	2 (7.69)	0.526
Occult obstruction type, *n* (%)	17 (25)	3 (11.54)	0.154
Pulmonary hypertension, *n* (%)	18 (26.47)	8 (30.77)	0.677
History of alcohol ablation, *n* (%)	3 (4.41)	0 (0)	0.665
History of septal myectomy, *n* (%)	1 (1.47)	0 (0)	1
History of radiofrequency ablation, *n* (%)	1 (1.47)	0 (0)	1
**Pre-operative medication**			
Calcium channel blockers, *n* (%)	15 (22.06)	8 (30.77)	0.380
Beta-blockers, *n* (%)	53 (77.94)	16 (61.54)	0.107
Diuretics, *n* (%)	12 (17.65)	5 (19.23)	0.907
Amiodarone, *n* (%)	22 (32.35)	11 (42.31)	0.366
**Anticoagulant drugs**			
Warfarin, *n* (%)	16 (23.53)	10 (38.46)	0.148
New oral anticoagulants, *n* (%)	5 (7.36)	3 (11.54)	0.518

*IQR, interquartile range; SD, standard deviation; EuroSCORE II, European System for Cardiac Operative Risk Evaluation II; NYHA, New York Heart Association*.

### Operative Data and Early Clinical Outcomes

Septal myectomy was performed in all patients, combined with the Cox-maze procedure in 68 (72.34%) patients, including left atrial ablation in 14 (14.89%) cases and bilateral ablation in 54 (57.45%) cases, and not combined with the Cox-maze procedure in 26 (27.66%) patients. All patients underwent left atrial appendectomy. Patients in the Cox-maze group had longer cardiopulmonary bypass time (178.50 [154.75, 197.00] vs. 95.00 [85.25, 128.00] min, *P* < 0.001) and aortic cross-clamp time (122.50 [103, 144.25] vs. 65.00 [53.50, 93.25] min, *P* < 0.001) than those in the no Cox-maze group; however, the groups did not differ in terms of mechanical ventilation time, post-operative hospital stay, and incidence of major complications (Table 2), and there was no stroke or thrombosis in the two groups. After we removed the patients who underwent left atrial ablation, the baseline data and post-operative results of the two groups were still consistent with those before (Supplementary Tables 4–7). Two patients in the Cox-maze group died during the post-operative hospital stay. One patient suffered from severe cardiac and renal insufficiency in the later period and died of multiple organ failure 37 days after surgery. The other patient suffered from severe renal insufficiency and died of malignant arrhythmia 2 weeks after surgery.

**Table 2:** Comparison of perioperative outcomes and early complications of patients in the Cox-maze and no Cox-maze group.

**Post-operative data**	**Cox-maze (*N* = 68) (%)**	**No Cox-maze (*N* = 26) (%)**	***P*-value**
CPB time (min), median (IQR)	178.50 (154.75, 197.00)	95.00 (85.25, 128.00)	<0.001
Aortic clamp time (min), median (IQR)	122.50 (103, 144.25)	65.00 (53.50, 93.25)	<0.001
Ventilation time (hour), median (IQR)	18 (15, 26)	18 (17, 26)	0.551
ICU stay (hour), median (IQR)	72 (48, 96)	72 (48, 120)	0.833
Length of stay (day), median (IQR)	11 (8, 14)	10 (7, 16)	0.629
**Concomitant procedures**			
Tricuspid annuloplasty, *n* (%)	20 (29.41)	5 (19.23)	0.318
Mitral valvuloplasty, *n* (%)	10 (14.71)	2 (7.70)	0.362
Mitral valve replacement, *n* (%)	4 (5.88)	1 (3.85)	0.694
Aortic valve replacement, *n* (%)	1 (1.47)	0 (0)	1
CABG, *n* (%)	7 (10.29)	1 (3.85)	0.316
Myocardial bridge release, *n* (%)	3 (4.41)	4 (15.38)	0.070
Resection of ventricular aneurysm, *n* (%)	2 (2.94)	0 (0)	0.932
Thrombectomy of left atrium, *n* (%)	1 (1.47)	0 (0)	1
**Complications**			
Perioperative death, *n* (%)	2 (2.94)	0 (0)	0.932
Pacemaker implantation, *n* (%)	3 (4.41)	0 (0)	0.665
Complete LBBB, *n* (%)	21 (30.88)	13 (50)	0.084
CRRT, *n* (%)	2 (2.94)	0 (0)	0.932
IABP, *n* (%)	1 (1.47)	0 (0)	1
Re-intubation, *n* (%)	1 (1.47)	0 (0)	1
Poor healing of surgical wound, *n* (%)	0 (0)	1 (3.85)	0.616
Pericardial effusion, *n* (%)	1 (1.47)	0 (0)	1

*CPB, cardiopulmonary bypass; CABG, coronary artery bypass grafting; LBBB, left bundle branch block; CRRT, continuous renal replacement therapy; IABP, intra-aortic balloon pump; IQR, interquartile range; ICU, intensive care unit*.

The pre-operative and post-operative echocardiographic data of patients in the Cox-maze and no Cox-maze groups are detailed in Supplementary Table 1. Patients in the Cox-maze group had slightly larger pre-operative left atrial diameters (51.09 ± 6.75 vs. 48.15 ± 6.99 mm, *P* = 0.07) than those in the no Cox-maze group, but this difference was not statistically significant.

### Follow-Up

The median (IQR) follow-up was 25 (6–48.5) months. One patient in the Cox-maze group died suddenly 2 months after the surgery. Three patients in the no Cox-maze group died, including one who died due to heart failure 3 months after surgery, one due to liver cancer 2 years after surgery, and one sudden death 1 year after surgery.

Compared with patients in the no Cox-maze group, those in the Cox-maze group had lower mid-term AF recurrence (*P* = 0.01). There were no significant differences in stroke and thrombotic events (*P* = 0.073) or other complications between the two groups (Table 3).

**Table 3:** Major adverse cardiovascular events of patients in the Cox-maze and no Cox-maze group during follow-up.

**Post-operative complications**	**Cox-maze (*N* = 66) (%)**	**No Cox-maze (*N* = 26) (%)**	***P*-value**
All-cause death, *n* (%)	1 (1.52)	3 (11.54)	0.108
Sudden cardiac death, *n* (%)	1 (1.52)	1 (3.85)	1
Death from heart failure, *n* (%)	0 (0)	1 (3.85)	0.275
Death from malignant tumor, *n* (%)	0 (0)	1 (3.85)	0.275
Hospitalization for AF occurrence, *n* (%)	4 (6.06)	4 (15.38)	0.280
Permanent pacemaker implantation, *n* (%)	4 (6.06)	2 (7.69)	1
Hospitalization for heart failure, *n* (%)	1 (1.52)	1 (3.85)	1
Stroke and thrombus, *n* (%)	0 (0)	2 (7.69)	0.073
Radiofrequency ablation, *n* (%)	3 (4.55)	1 (3.85)	1.00
AF recurrence, *n* (%)	15 (22.73)	13 (50)	0.010
NYHA functional class ≥III, *n* (%)	2 (3.03)	3 (11.54)	0.267

*NYHA, New York Heart Association; AF, atrial fibrillation*.

**Figure 1:**
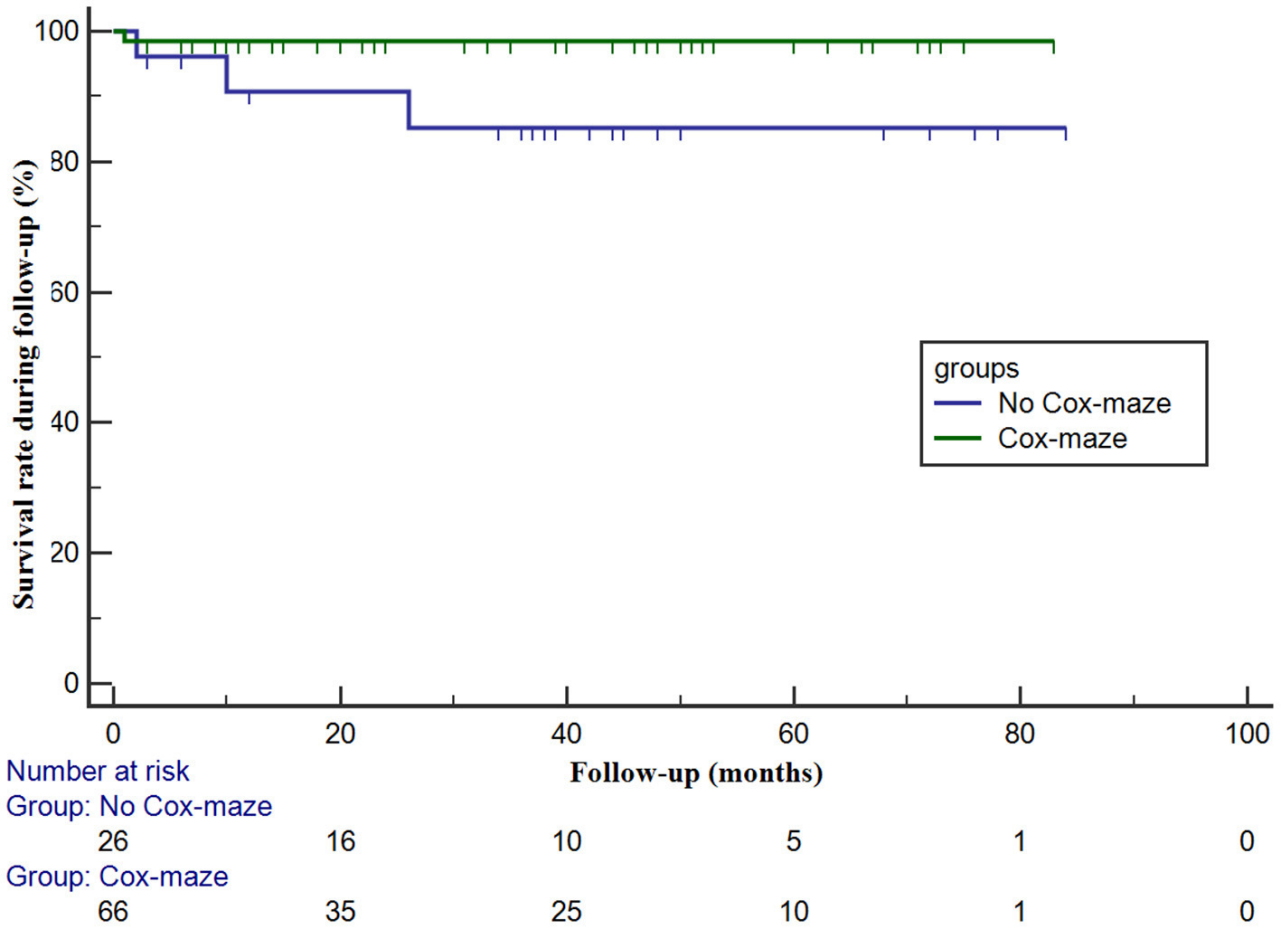
The survival in patients with hypertrophic obstructive cardiomyopathy according to whether the concomitant Cox-maze procedure was performed or not. The survival in patients with and without the Cox-maze procedure is different (log-rank statistic, 4.013; *P* = 0.046).

During the follow-up period, freedom from all-cause mortality after septal myectomy at 1, 3, and 5 years was 98.5 ± 1.5% each in the Cox-maze group, and 90.8 ± 6.3%, 85.1 ± 8.1%, and 85.1 ± 8.1%, respectively, in the no Cox-maze group. The Kaplan–Meier survival curves showed a lower survival in patients in the no Cox-maze group than in those in the Cox-maze group (*P* = 0.046, Figure 1).

### Atrial Fibrillation Recurrence Outcome

There were 28 cases of AF recurrence in all patients (Supplementary Table 2 and Table 3), including 15 in the Cox-maze group and 13 in the no Cox-maze group. Compared with patients with no AF recurrence, those with AF recurrence were significantly older [55 (47.25, 65) vs. 51.5 (39, 60.25), *P* = 0.027], had larger post-operative left atrial diameters (LADs) (45.30 ± 7.40 mm vs. 41.02 ± 6.51 mm, *P* = 0.009), larger post-operative LV end-diastolic diameters (48.78 ± 4.89 mm vs. 46.48 ± 4.76 mm, *P* = 0.042), and more likely to have chest pain (75 vs. 27.27%, *P* < 0.001). Among the 28 patients, 9 successfully recovered to SR after drug control (amiodarone), including 6 in the Cox-maze group and 3 in the no Cox-maze group.

**Figure 2:**
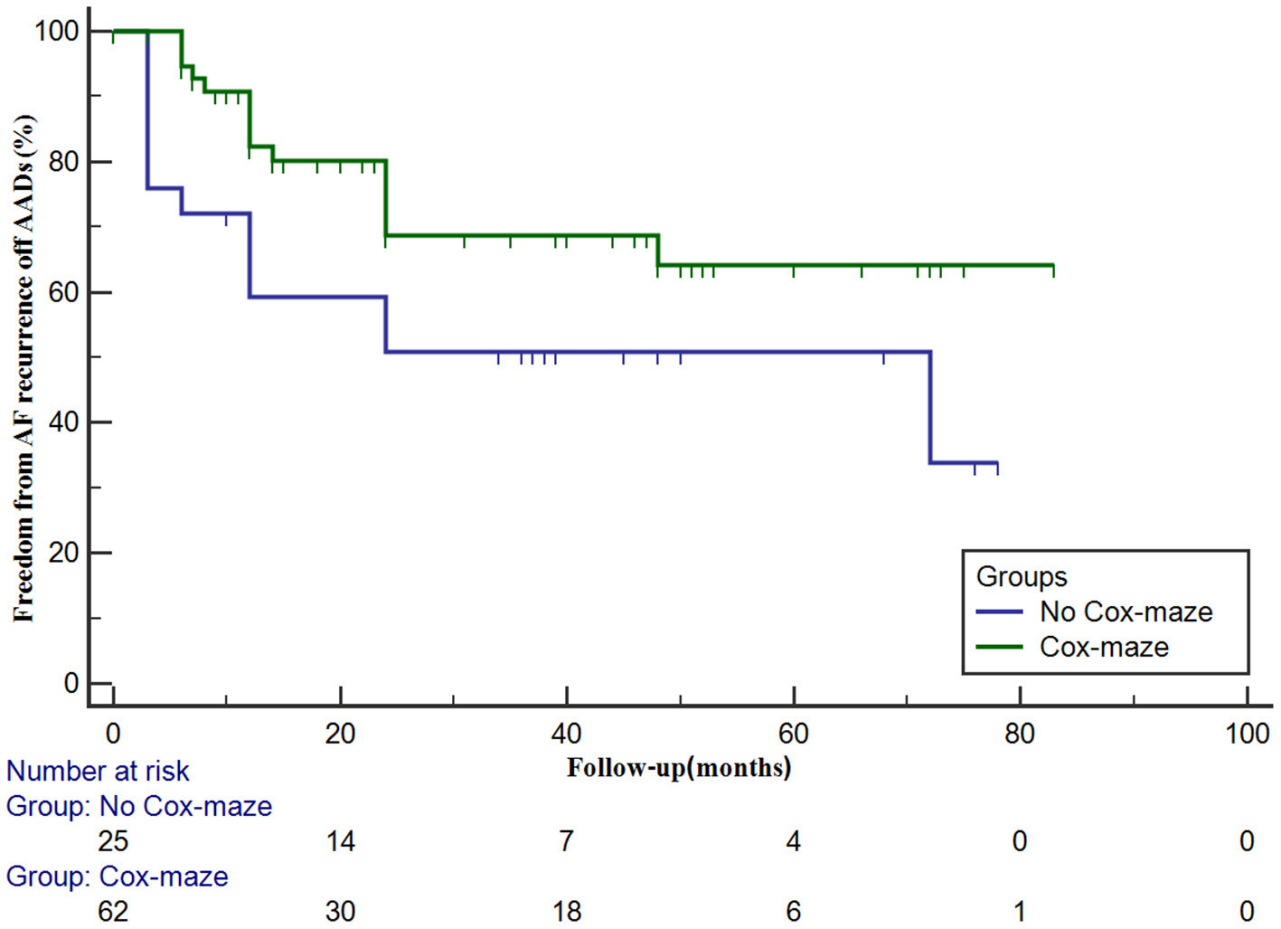
Freedom from atrial fibrillation (AF) recurrence off antiarrhythmic drugs (AADs) in patients with hypertrophic obstructive cardiomyopathy according to whether the concomitant Cox-maze procedure was performed or not. Freedom from AF recurrence off AADs in patients with and without the Cox-maze procedure is different (log-rank statistic, 4.199; *P* = 0.040).

**Figure 3:**
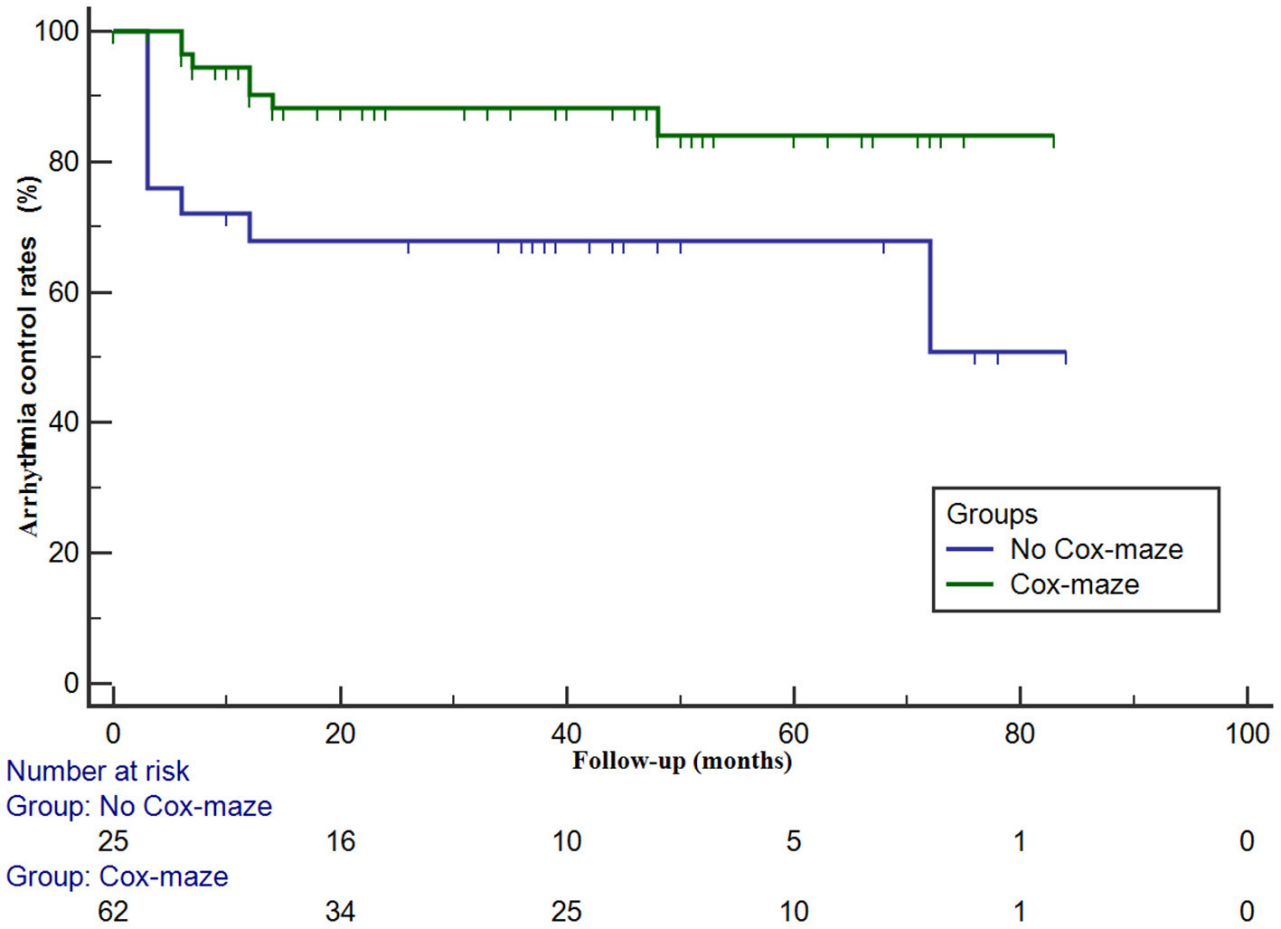
Arrhythmia control rates (including patients with successful AADs conversion) in patients with hypertrophic obstructive cardiomyopathy according to whether the concomitant Cox-maze procedure was performed or not. Arrhythmia control rates in patients with and without the Cox-maze procedure is different (log-rank statistic, 6.379; *P* = 0.012).

In the Cox-maze group, freedom from AF recurrence off antiarrhythmic drugs (AADs) was 90.7 ± 4.0% at 1 year and 68.7 ± 7.2% at 3 years. The 1- and 3-year arrhythmia control rates (including patients with successful AADs conversion) were 90.3 ± 4.1% and 88.2 ± 4.6%, respectively. In patients in the no Cox-maze group, freedom from AF recurrence off AADs was 59.3 ± 10.0% at 1 year and 50.8 ± 10.2% at 3 years. The arrhythmia control rates were 67.8 ± 9.4% and 67.8 ± 9.4% at 1 and 3 years, respectively. As presented in Figures 2, 3, the Kaplan–Meier survival curve showed a lower freedom from AF recurrence off AADs and arrhythmia control rate in patients in the no Cox-maze group than in the Cox-maze group (*P* = 0.040 and *P* = 0.012, respectively).

In the multivariate Cox proportional hazards regression analysis, after adjusting for confounding factors, it was found that the AF recurrence rate in patients who underwent the Cox-maze IV procedure was lower than that in patients who did not (hazard ratio, 0.141; 95% confidence interval [CI], 0.042–0.479; *P* = 0.002). Furthermore, post-operatively increased LADs (hazard ratio, 1.099; 95% CI, 1.024–1.179; *P* = 0.009) were associated with AF recurrence (Table 4).

**Table 4:** Predictors of AF recurrence following myectomy.

**Variables**	**Univariable model**		**Multivariable model**	
	**Hazard ratio (95% CI)**	***P*-value**	**Hazard ratio (95% CI)**	***P*-value**
Age	1.041 (1.005–1.078)	0.024	1.036 (0.996–1.077)	0.078
Male gender	1.103 (0.500–2.433)	0.808	1.571 (0.531–4.644)	0.414
Concomitant Cox-maze procedure	0.459 (0.212–0.995)	0.049	0.141(0.042–0.479)	0.002
Average BMI	1.077 (0.993–1.169)	0.073	1.013 (0.889–1.154)	0.849
History of cerebral infarction	2.587 (0.892–7.501)	0.080	1.471 (0.303–7.154)	0.632
Smoking history	2.262 (0.959–5.335)	0.062	1.557 (0.557–4.350)	0.398
Post-operative LADs	1.063 (1.008–1.063)	0.024	1.099 (1.024–1.179)	0.009
Post-operative LVEDDs	1.090 (1.006–1.090)	0.035	1.058 (0.947–1.181)	0.319
Paroxysmal AF	1.049 (0.478–2.299)	0.906		
Left atrial ablation	0.802 (0.272–2.366)	0.690		
Pre-operative LADs	1.019 (0.962–1.079)	0.524		
Pre-operative LVEDDs	1.063 (0.983–1.150)	0.125		

*BMI, body mass index; LADs, left atrial diameters; LVEDDs, left ventricular end-diastolic diameters; AF, atrial fibrillation*.

## Discussion

Our study showed that the mid-term survival of patients who underwent septal myectomy with the Cox-maze IV procedure was higher than that of patients who did not undergo the Cox-maze IV procedure. With regard to AF recurrence, the SR maintenance of patients who underwent the Cox-maze IV procedure was higher than that of patients who did not undergo the Cox-maze IV procedure.

There is a high incidence of AF in HOCM, and the related pathogenesis includes increased left atrial afterload caused by LV diastolic dysfunction, mitral regurgitation caused by SAM sign, atrial myopathy mainly manifested as atrial fibrosis, and atrial ischaemia due to microvascular dysfunction (6, 15). Many studies (6, 16, 17) have also documented that AF is associated with higher rates of heart failure, thromboembolism, and mortality. In a study of 4,248 patients with HOCM, the mortality of cardiovascular and non-cardiovascular diseases in patients with AF was higher than that in patients without AF (17). In our previous post-operative study of 472 patients with HOCM, AF was found to be an independent risk factor for post-operative cardiovascular death and cardiovascular events (18).

For patients with HOCM and AF, the curative effect of drug therapy and catheter ablation therapy is not satisfactory (6, 7), and there is no clear conclusion about the effect of septal myectomy combined with surgical ablation. It is still unclear whether isolated ventricular septectomy could reduce AF recurrence by reducing the peak LVOT gradients, thus avoiding surgical ablation. Our study showed that the mid-term survival of patients who underwent the Cox-maze IV procedure was higher than that of patients who did not undergo this procedure and the rates of mid-term AF recurrence were lower than those of patients without the Cox-maze IV procedure. During the follow-up period, the SR was restored in approximately half of the patients with AF after isolated septal myectomy. This also indicated that the reduction of LVOT gradients and mitral valve SAM sign partially eliminated AF recurrence, but some patients still had AF recurrence. Our results suggest that septal myectomy combined with surgical ablation can reduce AF recurrence and improve mid-term survival. A recent retrospective study (19) suggested that the maze procedure had no effect on mid-term survival in patients with HOCM complicated with AF. The time span of the patients in that study was very long, and the methods of the maze procedure varied. In addition, the literature does not follow up on the AF recurrence rate, and AF has a greater influence on the quality of life and cost of patients with HOCM.

Our study revealed that freedom from AF recurrence off AADs was 90.7 ± 4.0% at 1 year and 68.7 ± 7.2% at 3 years. Moreover, most patients underwent the biatrial Cox-maze IV procedure, and each ablation line was ablated six times; these results are similar to those of other recent studies. Boll et al. (10) described a series of 67 patients with symptomatic paroxysmal AF who underwent myectomy combined with the biatrial Cox-maze IV procedure. The reported freedom from recurrent symptomatic AF was 85% (95% CI, 73–92) at 1 year, 69% (95% CI, 55–79) at 3 years, and 64% (95% CI, 48–75) at 5 years. Subclinical AF and permanent AF were excluded from this study, and radiofrequency and cryoablation were used for surgical ablation. Another study (11) reported 45 patients with AF (26 paroxysmal AF/19 persistent AF) who underwent extended ventricular septal myomectomy and Cox-maze IV procedure with radiofrequency and cryotherapy, with 23.7 ± 1.3 months of follow-up. In this study, the rate of AF freedom was 89% (40 patients) at 1 year and 78% (35 patients) at 24 months, and each ablation line was ablated 8–10 times. According to Lapenna et al. (12), the SR maintenance rate of 31 patients with HOCM combined with persistent AF could reach 96 ± 3.5% and 80 ± 8.1% at 1- and 6-year arrhythmia control (maintenance of SR with or without AADs) after septal myectomy and surgical ablation. We also calculated the maintenance of the SR. The results showed that the 1- and 3-year arrhythmia control rates were 90.3 ± 4.1% and 88.2 ± 4.6%, respectively. Therefore, these limited studies have shown that active use of AADs can improve SR maintenance in patients with HOCM who underwent the Cox-maze IV procedure.

Previous studies (20, 21) have indicated that age, atrial diameter, hypertension, smoking, post-operative atrial tachycardia, and higher EuroSCORE are predictors of AF recurrence after ablation concomitant with cardiac operations; however, there is limited evidence from patients with HOCM combined with AF (11). Our study showed that patients with AF recurrence were older, had more symptoms of pre-operative chest pain, and had larger post-operative LADs and larger LV diameters. Further Cox regression analysis showed that post-operative LADs were associated with AF recurrence. Interestingly, compared with other studies (10, 20), our study confirms that pre-operative LADs were not associated with AF recurrence after surgery. The increases in post-operative LADs means that atrial remodeling is incomplete, which may continue to affect AF recurrence to a greater extent. Undoubtedly, our results suggest that the AF recurrence rate in patients with Cox-maze IV procedure was lower than that in patients without Cox-maze IV procedure, which confirms that the Cox-maze IV procedure should be actively performed in such patients to maintain post-operative SR.

### Study Limitations

This study has some limitations. This was a single-center retrospective control study, and the sample size was small, as it was limited by retrospective data; thus, it is difficult to perform meaningful statistical analysis. In view of the low incidence rate of HOCM and the small number of such operations, our total surgical volume was relatively high. Previous studies (10–12) on the surgical treatment of patients with HOCM and AF were mostly retrospective observational studies. Our study included patients with HOCM and AF who underwent isolated septal myectomy for various reasons in our center and were compared with patients who underwent the Cox-maze IV procedure at the same time, which further confirmed the necessity of the Cox-maze IV procedure for such patients. Another limitation is that we adopted different ablation strategies, including left atrial ablation and biatrial ablation, due to the experience of the surgeon and patients' conditions, we found that after we removed the patients who underwent left atrial ablation for statistics, the baseline data and post-operative results of the two groups were still consistent with those before. In addition, because cryoablation has not been approved in our country, we cannot add cryoablation energy to our ablation strategy. In the future, different energy types can be used for ablation, combined with the application of a catheter and surgical ablation to achieve better surgical results. Large-scale prospective studies are needed to confirm the effectiveness and safety of different ablation strategies in the future.

## Conclusion

In summary, our study confirmed that the Cox-maze IV procedure combined with septal myectomy improved mid-term survival and reduced mid-term AF recurrence in patients with HOCM and AF. The concomitant Cox-maze IV procedure is associated with a lower AF recurrence in patients with surgical HOCM and AF.

## Data Availability Statement

The original contributions presented in the study are included in the article/Supplementary Material, further inquiries can be directed to the corresponding author/s.

## Ethics Statement

The studies involving human participants were reviewed and approved by the ethics committee of Fuwai Hospital. The ethics committee waived the requirement of written informed consent for participation.

## Author Contributions

All authors listed have made a substantial, direct and intellectual contribution to the work, and approved it for publication.

## Conflict of Interest

The authors declare that the research was conducted in the absence of any commercial or financial relationships that could be construed as a potential conflict of interest.

## Publisher's Note

All claims expressed in this article are solely those of the authors and do not necessarily represent those of their affiliated organizations, or those of the publisher, the editors and the reviewers. Any product that may be evaluated in this article, or claim that may be made by its manufacturer, is not guaranteed or endorsed by the publisher.
