# Supplementary material for: Clinical Efficacy and Safety of Cox-Maze IV Procedure for Atrial Fibrillation in Patients With Hypertrophic Obstructive Cardiomyopathy
Source: Front Cardiovasc Med. 2021 Aug 2;8:720950. doi: 10.3389/fcvm.2021.720950 (PMC8365245; doi:10.3389/fcvm.2021.720950)
Supplement: Supplementary file 1 [file Data_Sheet_1.docx]

Supplementary Material

# Supplementary Data

**Supplemental Material 1:**

HOCM was diagnosed based on the presence of LV hypertrophy in the absence of other cardiac or systemic diseases that could cause LV hypertrophy. Septal myectomies were performed on patients whose symptoms were refractory to drug treatment and whose maximum LVOT gradients or midventricular gradients were ≥50 mmHg at rest or following physiologic provocation. The diagnostic criteria for AF were that AF attack (>30 s) could be accurately recorded by electrocardiography (ECG) or a Holter monitor, paroxysmal AF could stop within 7 days, persistent AF could last for more than 7 days, or the requirement of cardioversion. AF recurrence was defined as AF, atrial flutter, or atrial tachycardia > 30 s recorded by ECG or dynamic electrocardiogram 3 months after the operation. The criteria and surgical indications with regard to HOCM and AF were consistent with the 2011 American Heart Association/American College of Cardiology guidelines (1) and the 2014 European Society of Cardiology guideline (2). Because of the serious harm of AF in patients with HOCM, once AF was found in patients with HOCM who need surgery (all of them are treated with antiarrhythmic drugs), Cox-maze operation should be considered to restore sinus rate at the same time, unless the patients do not agree with concomitant Cox-maze operation.

**1.** Gersh BJ, Maron BJ, Bonow RO, et al. 2011 ACCF/AHA guideline for the diagnosis and treatment of hypertrophic cardiomyopathy: a report of the American College of Cardiology Foundation/American Heart Association Task Force on Practice Guidelines. *Circulation* 2011;124:e783-831.

**2.** Elliott PM, Anastasakis A, Borger MA, et al. 2014 ESC Guidelines on diagnosis and management of hypertrophic cardiomyopathy: the Task Force for the Diagnosis and Management of Hypertrophic Cardiomyopathy of the European Society of Cardiology (ESC). *Eur Heart J* 2014;35:2733-79.

**Supplemental Material 2:**

Extended septal myectomy: The hypertrophic ventricular septum that caused the SAM of the mitral valve and LVOT obstruction was resected. Resection along the longitudinal axis ranged from approximately 4 mm below the aortic ring to the apex of the LV and beyond the bases of the papillary muscles. Along the short axis, the myectomy began rightward to the nadir of the right aortic cusp, advanced to the left, and terminated near the mitral anterior commissure. Hypertrophy of the LV anterior free wall that caused LVOT narrowing also required resection. Furthermore, the anomalous chordal attachments that affected the LVOT were excised.

**Supplemental Material 3:**

Ablation lines: Through the median sternal incision, cardiopulmonary bypass was routinely established, and right atrium ablation was performed during parallel circulation. The right atrial ablation route included the ablation line between the superior and inferior venae cavae, the ablation line between the right atrial appendage and tricuspid annulus, the ablation line between the right atrial appendage and right atriotomy, and the ablation line across the right atrial free wall toward the atrioventricular groove near the acute margin of the heart (crista lesion). The left atrial appendage was excluded using an external double-layer 4/0 polypropylene suture after aortic cross-clamp. The left atrial ablation route included the ablation line of the left and right pulmonary vein openings, the ablation line between the right and left pulmonary veins (one on the roof and the other on the floor of the left atrium forming a ‘box lesion’), the ablation line between the left upper pulmonary vein orifice and left atrial appendage, and the ablation line between the right lower pulmonary vein isolation ring and posterior mitral valve ring, and the Marshall ligament was removed. Monopolar pens were used in mitral and tricuspid lines. A monopolar pen was used to ablate the coronary vein from the ostium to the wall of coronary vein, with a length of 25-30mm. Temporary epicardial pacing leads were placed after all the operations.

# Supplementary Tables

**Supplemental Table 1**: Pre- and postoperative echocardiography characteristics of patients in the Cox-maze and no Cox-maze group

| Variables | Cox-maze  (N=68) (%) | No Cox-maze  (N=26) (%) | *P*-value |
| --- | --- | --- | --- |
| Preoperative data |  |  |  |
| IVS (mm), median (IQR) | 18 (17, 22) | 21 (17, 24) | 0.187 |
| LADs (mm), mean ± SD | 51.09±6.75 | 48.15±6.99 | 0.073 |
| LVEDDs (mm), mean ± SD | 42.75±4.50 | 43.42±5.51 | 0.544 |
| LVEF (%), mean ± SD | 69.75±5.71 | 71.30±5.30 | 0.189 |
| Peak LVOT gradient (mmHg), median (IQR) | 64 (55, 81) | 74(56, 97) | 0.193 |
| Moderate or severe MR, n (%) | 31 (45.59) | 16 (61.54) | 0.249 |
| Postoperative data |  |  |  |
| IVS (mm), median (IQR) | 13 (11, 15) | 13 (11, 15) | 0.957 |
| LADs (mm)，mean ± SD | 41.19±10.14 | 39.05±6.94 | 0.191 |
| LVEDDs (mm), mean ± SD | 46.76±7.68 | 46.33±4.26 | 0.808 |
| LVEF (%), mean ± SD | 62.73±5.96 | 59.00±13.84 | 0.478 |
| Residual LVOT gradients (mmHg), median (IQR) | 10 (7.8, 15) | 13 (10.5, 17) | 0.060 |
| Moderate MR (%), n (%) | 5 (7.35) | 2 (7.69) | 0.955 |

IVS: Interventricular septum; LADs: Left atrial diameters; LVEDDs: Left ventricular end-diastolic diameters; LVEF: Left ventricular ejection fraction; LVOT: left ventricular outflow tract; MR: mitral regurgitation; IQR: interquartile range; SD: standard deviation.

**Supplementary Table 2:** Baseline characteristics, preoperative and postoperative clinical variables of patients with and without AF recurrence

| Characteristics | No AF recurrence  (N=66) (%) | AF recurrence  (N=28) (%) | *P*-value |
| --- | --- | --- | --- |
| Age (year), median (IQR) | 51.5 (39, 60.25) | 55 (47.25, 65) | 0.027 |
| Gender (males), n (%) | 43 (63.23) | 11 (42.31) | 0.066 |
| Body mass index (kg/m^2^), mean ± SD | 25.90 ± 3.59 | 27.17 ± 4.49 | 0.158 |
| NYHA functional class III/IV, n (%) | 52 (76.47) | 20 (76.92) | 1.00 |
| Paroxysmal, n (%) | 37 (56.06) | 15 (53.57) | 0.824 |
| Symptoms |  |  |  |
| Dyspnoea, n (%) | 60 (90.91) | 23 (82.14) | 0.227 |
| Chest pain, n (%) | 18 (27.27) | 11 (75) | <0.001 |
| Syncope, n (%) | 14 (21.21) | 5 (17.86) | 0.711 |
| Palpitation, n (%) | 20 (30.30) | 8 (28.57) | 0.867 |
| Comorbidities |  |  |  |
| Hypertension, n (%) | 20 (30.30) | 13 (46.43) | 0.134 |
| Hyperlipidemia, n (%) | 14 (21.21) | 7 (25) | 0.635 |
| Diabetes mellitus, n (%) | 5 (7.58) | 2 (7.14) | 0.942 |
| Coronary atherosclerotic heart disease, n (%) | 5 (7.58) | 2 (7.14) | 0.942 |
| Myocardial bridge, n (%) | 6 (9.09) | 1 (3.57) | 0.615 |
| History of cerebral infarction, n (%) | 1 (1.52） | 4 (14.28） | 0.043 |
| Smoking, n (%) | 6 (9.09) | 7 (25 ) | 0.041 |
| Preoperative echocardiography |  |  |  |
| LVEDDs (mm), mean ± SD | 42.41 ± 4.98 | 44.18 ± 4.08 | 0.101 |
| LADs (mm), mean ± SD | 49.77 ± 6.47 | 51.46 ± 7.84 | 0.280 |
| IVS (mm), median (IQR) | 18 (16.5, 23) | 19.5 (17, 22) | 0.433 |
| LVEF (%), mean ± SD | 70.07 ± 5.91 | 70.60 ± 4.96 | 0.677 |
| Peak LVOT gradient (mmHg), median (IQR) | 68 (56.75, 88.25) | 61 (53.5, 76.5) | 0.213 |
| Postoperative echocardiography |  |  |  |
| LVEDDs (mm), mean ± SD | 46.48 ± 4.76 | 48.78 ± 4.89 | 0.042 |
| LADs (mm), mean ± SD | 41.02 ± 6.51 | 45.30 ± 7.40 | 0.009 |
| IVS (mm), median (IQR) | 13(11.25, 15) | 13 (11, 15) | 0.826 |
| LVEF (%), mean ± SD | 62.43 ± 5.42 | 60.57 ± 13.08 | 0.623 |
| Peak LVOT gradient (mmHg), median (IQR) | 12 (9, 15.5) | 10 (7, 16) | 0.525 |
| CPB (min), median (IQR) | 163 (131, 186) | 158 (96, 193) | 0.413 |
| Aortic cross-clamp time (min), median (IQR) | 117 (95, 133) | 108 (69, 136) | 0.423 |
| Ventilation time (hour), median (IQR) | 18 (15, 26) | 17.5 (16, 26.75) | 0.975 |
| ICU stay (hour), median (IQR) | 72 (48, 114) | 60 (30, 114) | 0.445 |
| Length of stay (day), median (IQR) | 11 (8, 15) | 9.5 (7, 13) | 0.240 |

IVS: Interventricular septum; LADs: Left atrial diameters; LVEDDs: Left ventricular end-diastolic diameters; LVEF: Left ventricular ejection fraction; LVOT: left ventricular outflow tract; ICU, intensive care unit; NYHA, New York Heart Association; CPB: Cardiopulmonary bypass time time; IQR: interquartile range; SD: standard deviation;

**Supplementary Table 3**: Comparison of early and complications of patients with and without AF recurrence.

| Postoperative complications | No AF recurrence  (N=66) (%) | AF recurrence  (N=28) (%) | *P*-value |
| --- | --- | --- | --- |
| Early complications |  |  |  |
| Perioperative death, n (%) | 2 (3.03) | 0 (0) | 1 |
| CRRT, n (%) | 2 (3.03) | 0 (0) | 1 |
| IABP, n (%) | 1 (1.52) | 0 (0) | 1 |
| Re-intubation, n (%) | 1 (1.52) | 0 (0) | 1 |
| Poor healing of surgical wound, n (%) | 1 (1.52) | 0 (0) | 1 |
| Pericardial effusion, n (%) | 1 (1.52) | 0 (0) | 1 |
| Follow-up complications |  |  |  |
| All-cause death, n (%) | 3 (4.55 ) | 1 (3.57) | 1 |
| Hospitalization for heart failure, n (%) | 0 (0) | 2 (7.14) | 0.086 |
| Stroke and thrombus, n (%) | 0 (0) | 2 (7.14) | 0.086 |

CRRT: continuous renal replacement therapy; IABP: Intra-aortic balloon pump.

**Supplementary Table 4**: Baseline characteristics and preoperative clinical variables of patients in the Cox-maze and no Cox-maze group.(without left atrial ablation)

| Characteristics | Cox-maze  (N=54) (%) | No Cox-maze  (N=26) (%) | *P*-value |
| --- | --- | --- | --- |
| Age (year), median (IQR) | 53 (42, 61.25) | 54 (42, 64) | 0.770 |
| Gender (males), n (%) | 30 (55.56) | 11 (42.31) | 0.034 |
| Body mass index (kg/m^2^), mean ± SD | 26.16 ± 3.43 | 26.39 ± 5.15 | 0.816 |
| NYHA functional class III/IV, n (%) | 40 (74.07) | 20 (76.92) | 1.00 |
| EuroSCORE II, mean ± SD | 2.85 ± 0.96 | 1.77 ± 1.42 | 0.001 |
| Paroxysmal, n (%) | 25 (46.30) | 16 (61.54) | 0.299 |
| Symptoms |  |  |  |
| Dyspnoea, n (%) | 50 (90.74) | 20 (76.92) | 0.049 |
| Chest pain, n (%) | 15 (27.78) | 11 (42.31) | 0.194 |
| Syncope, n (%) | 12 (22.22) | 2 (7.69) | 0.198 |
| Palpitation, n (%) | 19 (35.19) | 8 (30.77) | 0.919 |
| Comorbidities |  |  |  |
| Hypertension, n (%) | 21 (38.89) | 7 (26.92) | 0.584 |
| Hyperlipidemia, n (%) | 12 (22.22) | 9 (34.62) | 0.238 |
| Diabetes mellitus, n (%) | 3 (5.56) | 1 (3.85) | 1 |
| Coronary atherosclerotic heart disease, n (%) | 5 (9.26) | 1 (3.85) | 0.683 |
| Myocardial bridge, n (%) | 3 (5.56) | 4 (15.38) | 0.301 |
| History of cerebral infarction, n (%) | 2 (3.70） | 2 (7.69） | 0.827 |
| Occult obstruction type, n (%) | 12 (22.22) | 3 (11.54) | 0.400 |
| Pulmonary hypertension, n (%) | 13(24.07) | 8 (30.77) | 0.524 |
| History of alcohol ablation, n (%) | 2 (3.70) | 0 (0) | 1 |
| History of septal myectomy, n (%) | 1 (1.85) | 0 (0) | 1 |
| History of radiofrequency ablation, n (%) | 1 (1.85) | 0 (0) | 1 |
| Preoperative medication |  |  |  |
| Calcium channel blockers, n (%) | 12 (22.22) | 8 (30.77) | 0.408 |
| Beta-blockers, n (%) | 44 (81.48) | 16 (61.54) | 0.054 |
| Diuretics, n (%) | 9 (16.67) | 5 (19.23) | 0.777 |
| Amiodarone, n (%) | 20 (37.04 ) | 11 ( 42.31) | 0.650 |
| Anticoagulant drugs |  |  |  |
| Warfarin, n (%) | 14 (25.93) | 10 (38.46) | 0.572 |
| New oral anticoagulants, n (%) | 4 (7.41 ) | 3 (11.54) | 0.540 |

IQR: interquartile range; SD: standard deviation; NYHA: New York Heart Association.

**Supplementary Table 5**: Comparison of perioperative outcomes and early complications of patients in the Cox-maze and no Cox-maze group (without left atrial ablation)

| Postoperative data | Cox-maze  (N=54) (%) | No Cox-maze  (N=26) (%) | *P*-value |
| --- | --- | --- | --- |
| CPB time (min), median (IQR) | 179 (155.75, 200.50) | 95.00 (85.25, 128.00) | <0.001 |
| Aortic cross-clamp time (min), median (IQR) | 125.50 (102.25, 146.75) | 65.00 (53.50, 93.25) | <0.001 |
| Ventilation time (hour), median (IQR) | 18 (15, 35.50) | 18 (17, 26) | 0.718 |
| ICU stay (hour), median (IQR) | 72 (48, 120) | 72 (48, 120) | 0.863 |
| Length of stay (day), median (IQR) | 10.5(8, 14) | 10 (7, 16) | 0.598 |
| Concomitant procedures |  |  |  |
| Tricuspid annuloplasty, n (%) | 14 (25.93) | 5 (19.23) | 0.510 |
| Mitral valvuloplasty, n (%) | 7 (12.96) | 2 (7.70) | 0.485 |
| Mitral valve replacement, n (%) | 3 (5.56) | 1 (3.85) | 1 |
| Aortic valve replacement, n (%) | 1 (1.85) | 0 (0) | 1 |
| CABG, n (%) | 5 (9.26) | 1 (3.85) | 0.683 |
| Myocardial bridge release, n (%) | 3 (5.56) | 4 (15.38) | 0.301 |
| Resection of ventricular aneurysm, n (%) | 1 (1.85) | 0 (0) | 1 |
| Thrombectomy of left atrium, n (%) | 1 (1.85) | 0 (0) | 1 |
| Complications |  |  |  |
| Perioperative death, n (%) | 2 (3.70) | 0 (0) | 1 |
| Pacemaker implantation, n (%) | 3 (5.56) | 0 (0) | 0.547 |
| Complete LBBB, n (%) | 18 (33.33) | 13 (50) | 0.152 |
| CRRT, n (%) | 1 (1.85) | 0 (0) | 1 |
| IABP, n (%) | 1 (1.85) | 0 (0) | 1 |
| Re-intubation, n (%) | 1 (1.85) | 0 (0) | 1 |
| Poor healing of surgical wound, n (%) | 0 (0) | 1 (3.85) | 0.325 |
| Pericardial effusion, n (%) | 1 (1.85) | 0 (0) | 1 |

CPB: cardiopulmonary bypass; CABG: coronary artery bypass grafting; LBBB: left bundle branch block; CRRT: continuous renal replacement therapy; IABP: Intra-aortic balloon pump; IQR: interquartile range; ICU, intensive care unit.

**Supplementary Table 6**: Major adverse cardiovascular events of patients in the Cox-maze and no Cox-maze group during follow-up (without left atrial ablation)

| Postoperative complications | Cox-maze  (N=52) (%) | No Cox-maze  (N=26) (%) | *P*-value |
| --- | --- | --- | --- |
| All-cause death, n (%) | 1 (1.92 ) | 3 (11.54) | 0.189 |
| Sudden cardiac death, n (%) | 1 (1.92) | 1 (3.85) | 1 |
| Death from heart failure, n (%) | 0 (0) | 1 (3.85) | 0.333 |
| Death from malignant tumor, n (%) | 0 (0) | 1 (3.85) | 0.333 |
| Hospitalization for AF occurrence, n (%) | 4 (7.69) | 4 (15.38) | 0.509 |
| Permanent pacemaker implantation, n (%) | 4 (7.69) | 2 (7.69) | 1 |
| Hospitalization for heart failure, n (%) | 1 (1.92) | 1 (3.85) | 1 |
| Stroke and thrombus, n (%) | 0 (0) | 2 (7.69) | 0.108 |
| Radiofrequency ablation, n (%) | 2 (3.85 ) | 1 (3.85) | 1 |
| AF recurrence , n (%) | 10 (19.23) | 13 (50) | 0.005 |
| NYHA functional class ≥III, n (%) | 1 (1.92) | 3 (11.54) | 0.189 |

NYHA, New York Heart Association; AF: trial fibrillation.

**Supplementary Table 7**: Predictors of AF recurrence following myectomy (without left atrial ablation)

| variables | Univariable model | | Multivariable model | |
| --- | --- | --- | --- | --- |
|  | Hazard ratio (95% CI) | *P*-value | Hazard ratio (95% CI) | *P*-value |
| Age | 1.033 (0.997-1.071) | 0.070 | 1.039 (0.990-1.092) | 0.121 |
| Male gender | 1.111 (0.489-2.525) | 0.801 | 1.873 (0.569-6.167) | 0.302 |
| Concomitant Cox-maze procedure | 0.454 (0.195-1.053) | 0.065 | 0.150(0.038-0.591) | 0.007 |
| Average BMI | 1.080 (0.995-1.172) | 0.066 | 1.074 (0.944-1.222) | 0.279 |
| History of cerebral infarction | 3.991 (0.833-19.125) | 0.083 | 1.386 (0.258-7.441) | 0.704 |
| Preoperative LADs | 1.094 (0.993-1.205) | 0.069 | 0.993 (0.905-1.088) | 0.873 |
| Postoperative LADs | 1.147 (1.034-1.273) | 0.010 | 1.105 (1.017-1.200) | 0.018 |
| Postoperative LVEDDs | 1.152 (1.013-1.310) | 0.031 | 1.040 (0.904-1.197) | 0.583 |
| Paroxysmal AF | 1.097 (0.474-2. 539) | 0.828 |  |  |
| Preoperative LVEDDs | 1.104 (0.966-1.262) | 0.146 |  |  |

BMI: body mass index; LADs: Left atrial diameters; LVEDDs: Left ventricular end-diastolic diameters; AF: atrial fibrillation
